# Widely Targeted Metabolomic Analysis Provides New Insights into the Effect of Rootstocks on Citrus Fruit Quality

**DOI:** 10.3390/metabo14040242

**Published:** 2024-04-21

**Authors:** Min Wang, Yang Chen, Shuang Li, Jianjun Yu, Lei Yang, Lin Hong

**Affiliations:** 1 Fruit Tree Research Institute, Chongqing Academy of Agricultural Sciences, Chongqing 401329, China; wm950918@126.com (M.W.); sclishuang61@163.com (S.L.); jerksion@163.com (J.Y.); 2 Key Laboratory of Evaluation and Utilization for Special Crops Germplasm Resource in the Southwest Mountains, Ministry of Agriculture and Rural Affairs, Chongqing Academy of Agricultural Sciences, Chongqing 401329, China; lixianchy2008@163.com; 3 Biotechnology Research Institute, Chongqing Academy of Agricultural Sciences, Chongqing 401329, China

**Keywords:** rootstock, late-maturing hybrid mandarin varieties, fruit quality, UPLC-MS/MS, metabolomics

## Abstract

The use of different rootstocks has a significant effect on the content of flavor components and overall fruit quality. However, little information is available about the metabolic basis of the nutritional value of citrus plants. In this study, UPLC-MS/MS (ultra-performance liquid chromatography-tandem mass spectrometry) was performed to analyze the metabolites of three late-maturing hybrid mandarin varieties (‘Gold Nugget’, ‘Tango’ and ‘Orah’) grafted on four rootstocks (‘Trifoliate orange’, ‘Carrizo citrange’, ‘Red tangerine’ and ‘Ziyang Xiangcheng’). A total of 1006 metabolites were identified through OPLS-DA (Orthogonal Partial Least Squares-Discriminant Analysis) analysis. KEGG (Kyoto Encyclopedia of Genes and Genomes) analysis revealed the most critical pathways among the different pathways associated with genes grafted on the four rootstocks that were differentially activated, including tryptophan metabolism and sphingolipid metabolism in ‘Gold Nugget’; tryptophan metabolism, phenylpropanoid biosynthesis and sphingolipid metabolism in ‘Tango’; and pantothenate and CoA biosynthesis- and photosynthesis-related biosynthesis in ‘Orah’. A considerable difference between the different rootstocks was also observed in the accumulation of lipids, phenolic acids and flavonoids; further analysis revealed that the rootstocks regulated specific metabolites, including deacetylnomylinic acid, sudachinoid A, amoenin evodol, rutaevin, cyclo (phenylalanine-glutamic acid), cyclo (proline-phenylalanine), 2-hydroxyisocaproic acid, and 2-hydroxy-3-phenylpropanoic acid. The results of this study provide a useful foundation for further investigation of rootstock selection for late-maturation hybrid mandarin varieties.

## 1. Introduction

Hybrid citrus cultivars have become the most popular fresh citrus varieties because of their variety of characteristics, such as ease of peeling, rich flavor, and seedlessness. There are many kinds of hybrid mandarins, such as ‘Orah’, ‘Beni-Madonna’, ‘Chunjian’, ‘Buzhihuo’, and ‘W·Murcott’. Recently, ‘Gold Nugget’, ‘Tango’, and ‘Orah’, three late-maturing hybrid mandarins, have been recently introduced and developed in China as good market sprospects. The ‘Gold Nugget’ is a hybrid variety of the ‘Wilking’ and ‘Kincy’ mandarins [1]. Tango was induced by irradiation of the ‘W. Murcott’ [2]. ’Orah’ was obtained from the hybrid of the ‘Temple’ tangor and ‘Dancy’ mandarin [3]. Moreover, the mature periods of these plants are mostly from February to March, which is beneficial for adjusting the citrus industrial structure in Chongqing.

Grafting is an ancient, traditional horticulture technique used for improving crop qualities [4,5,6]. The biosynthesis of secondary metabolites is known to be affected by a variety of factors, with grafting playing a significant role. Grafted plants exhibit variations in metabolite compositions compared to non-grafted plants [7,8,9]. Rootstocks used for grafting of scion cultivars have obvious influences on tree growth, fruit quality, and yield, and on plant responses to biotic and abiotic stresses [10,11]. ‘Trifoliate orange’ [*Poncirus trifoliate* (L.) Raf.], Pt, ‘Carrizo citrange’ (*Citrus sinensis* Osb. × *P. trifoliate* Raf), ‘Red tangerine’ (*Citrus reticulata* Blanco cv. Red tangerine), and ‘Ziyang Xiangcheng’ (*Citrus junos* Sieb. ex Tanaka) are widely used as citrus rootstock resources in Southwest China. The most commonly used rootstock is trifoliate orange, which has excellent cold-, foot rot-, and tristeza virus- –resistance characteristic [10]. Several studies have shown that these rootstocks have excellent resistance and have significant effects on the growth process and fruit quality [12,13,14].

Metabolites are related to the phenotypes of organisms and directly reflect changes in metabolic processes [15,16,17]. Metabolomics play an important role in plant biology and applied biotechnology [18]. Qualitative and quantitative analyses of metabolites can be used to understand the composition, structure, synthesis pathway, and function of related genes to determine plant metabolic pathways. In recent years, ultra-performance liquid chromatography (UPLC)-tandem mass spectrometry (MS)/tandem mass spectrometry (MS) has been highly convenient for the study of plant metabolomics, as it has significantly improved the speed, sensitivity and separation rate of chromatographic analysis (Table A1 contains an abbreviation comparison table) [19,20]. Metabolomic analysis has been extensively applied to plants, such as orange [21], apple, peach [22], jujube [23], and loquat [24].

The metabolite composition depends on the cultivar and the plant species, which is useful for plant breeders [25]. Although there are many reports on the effects of different rootstocks on the fruit matter quality of a certain citrus cultivar and the efficacy of rootstock–spike combinations for different citrus cultivars, studies on the effects of rootstocks on the fruit matter quality of different citrus cultivars are rare. Fruits are among the most abundant metabolites in plant organs, so there are a large number of metabolites in their metabolic supplements. These metabolites change the taste and flavor of plants, have nutritional or medicinal properties [22], and even have plant defense effects against biological and abiotic stresses. Previous studies have shown that rootstock can influence the metabolic response of sweet orange trees to Huanglongbing (HLB) [26]. Rootstocks can affect the composition of the roots and leaves of young navel orange and pummelo trees [27]. However, the effect of rootstock use on metabolites in late-maturation mandarin varieties has not been determined. Here, we examined the fruit metabolites of three late-maturing scion cultivars on four rootstocks. The main purpose of this study was to explore the effects of four different rootstocks on fruit quality and metabolites and to provide a reference for rootstock selection and breeding improvement for late-maturing mandarin varieties.

## 2. Materials and Methods

### 2.1. Plant Materials and Treatments

**Figure 1:**
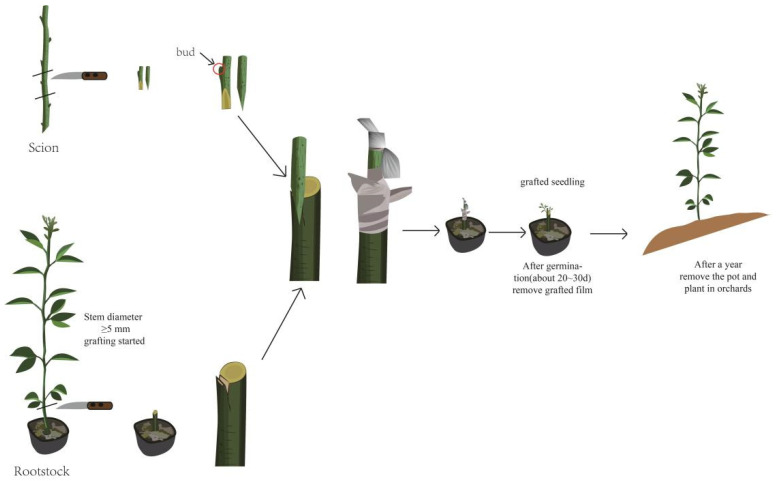
Grafting diagram. Fifteen fruits taken from each variety on different rootstocks were collected for fruit quality analysis at the fruit maturation stage. Fruit weights were acquired using an electronic balance. Fruit height and transverse diameter were measured by a digital scale. The fruit index refers to the ratio of the longitudinal diameter to the transverse diameter of the fruit. The fruit was cut in half on the equator, and the rind thickness was measured by a digital Vernier caliper. The total soluble solids (TSS) content was determined with a digital refractometer (PAL-1, ATAGO, Japan). The titratable acidity (TA) was measured via titration with 0.1 mol/L NaOH solution using phenolphthalein as the indicator. The ascorbic acid (vitamin C, Vc) content was measured using 0.2 mL of fruit juice and 1.8 mL of oxalic acid and 2,6-dichlorophenol indophenol as a dye. Each set contained three replicates. All the statistical analyses were performed using SPSS 18.0 software (*p* < 0.05).

In March 2016, four types of rootstock plants (those with a stem diameter ≥ 5 mm) and three scion varieties with buds were taken for grafting. The four types of rootstock used were ‘Trifoliate orange’ (designated 1), ‘Carrizo Citange’ (designated 2), ‘Red tangerine’ (designated 3), and ‘Ziyang Xiangcheng’ (designated 4). The three scion varieties were ‘Gold Nugget’ (designated A), ‘Tango’ (designated B), and ‘Orah’ (designated C). There weas a total of 12 combinations, each of which was grafted with 12 trees, and each tree was grafted with a bud. The detailed process of grafting is shown in Figure 1. After grafting, the grafting interface was wrapped with grafting film to maintain humidity. After 20–30 days, the grafting interface germinated. After germination, the grafting film was removed, and the plants were cultured in pots until March 2017. In 2017, the scion plants were removed from the pots and planted at the Jiangjin Citrus Variety Test Base, Institute of Fruit, Chongqing Academy of Agricultural Sciences, Chongqing, China (longitude, 29°22 N; latitude, 106°30 E; altitude, 290 m), and the scion plants yielded normal results in 2019. The experiments were carried out from 2020 to 2022. For each combination, nine trees with consistent growth were selected as sample trees, with each of the three trees being a biological repeat, for a total of three replicates. Five fruits were taken from each tree during the fruit ripening period for basic quality measurement, with a total of 15 fruits per replicate. After the determination of fruit quality was completed, the juice of three duplicate samples was mixed and collected in a 50 ml centrifuge tube. After freezing in liquid nitrogen, the samples were placed in an ultralow temperature refrigerator for UPLC-MS/MS analysis.

### 2.2. Agents and Instrument

Methanol (HPLC grade, purity ≥ 99.9%) and acetonitrile (HPLC grade, purity ≥ 99.9%) were purchased from Merck Company (Darmstadt, Germany). Standard 2-chlorophenylalanine (HPLC grade, purity ≥ 99.5%) and dimethyl sulfoxide (HPLC grade, purity ≥ 99.7%) were purchased from Xili Company (Shanghai, China) and Sigma-Aldrich (St. Louis, MI, USA), respectively.

### 2.3. Sample Preparation

All the samples were subjected to freeze drying equipment for vacuum freeze drying and ground into powder with a grinding instrument at 30 Hz for 90 s. A quantity of 100 mg powder was removed, dissolved in 1.2 mL of 70% methanol extract and vortexed once every 30 min for 30 s, which was performed 6 times. Then, we placed the sample overnight in a 4 °C refrigerator. The sample was centrifuged (12000 r/min, 4 °C) for 10 min, and the supernatant was filtered by microfiltration (0.22 μm) and stored in an injection bottle for UPLC-MS/MS analysis.

### 2.4. UPLC-MS/MS Analysis

Metabolic analysis was performed using an ultraperformance liquid chromatography (UPLC) instrument (SHIMADZU Nexera X2, https://www.shimadzu.com.cn/, accessed on 15 May 2023) and tandem mass spectrometry (MS-MS) (Applied Biosystems 4500 Q TRAP, http://www.appliedbiosystems.com.cn/, accessed on 15 May 2023). The analytical conditions were as follows: an Agilent SB-C18 (1.8 µm, 2.1 mm×100 mm) was used. The mobile phases used were mobile phase A (pure water with 0.1% formic acid) and mobile phase B (acetonitrile with 0.1% formic acid). The elution gradient was as follows: 95% A 5% B at 0 min, 5% A 95% B at 9 min, maintained for 1 min, 5% B at 10–11 min and maintained for 3 min. The flow rate was maintained at 0.35 mL/min, and the injection volume was 4 μL. The column oven temperature was set to 40 °C.

The mass spectrometry procedure was as follows: LIT (linear ion trap) and QQQ (triple quadrupole) scanning were performed with an AB4500 Q TRAP UPLC/MS/MS system equipped with an ESI (electrospray ionization) Turbo Ion Spray Interface, which was controlled by Analyst 1.6.3 software in two modes, one with positive ions and one with negative ions.

The ESI source operating parameters were as follows: ion source, turbine spray; source temperature, 550 °C; ion spray voltage, 5000 V; and negative ion mode, −4500 V. The ion source gases GSI (ion source gas I) and GSII (ion source gas II) and the curtain gas (CUR) were set to 50, 60 and 25 psi, respectively, and high collision-induced ionization parameters were set. The instrument was tuned and calibrated with 10 and 100 µmol/L polypropylene glycol solution in the QQQ and LIT modes, respectively. A QQQ scan was performed using MRM (multiple reaction monitoring) mode, and the collision gas (nitrogen) was set to the appropriate medium. Through further optimization of DP (declustering potential) and CE (collision energy), the DP and CE of each MRM ion pair were completed. According to the metabolites eluted in each period, a special MRM ion pair was detected in each period.

All omic data were deposited in MetaboLights (https://www.ebi.ac.uk/metabolights (accessed on 15 May 2023), MTBLS9002), EMBL—European Bioinformatics Institute, Cambridge, UK.

### 2.5. Statistical Analysis

Hierarchical cluster analysis (HCA) was performed to determine the accumulation patterns of metabolites via the built-in statistical prcomp function of R. software (4.3.0), setting the prcomp function parameter scale = true, indicating that the data were subjected to unit variance scaling (UV). Principal component analysis (PCA) was performed with R software (4.3.0). Finally, FC ≥ 2 or FC ≤ 0.5 was used for screening differentially abundant metabolites. A Venn diagram was constructed to screen for differentially abundant metabolites among the three comparison groups. The differentially abundant metabolites were annotated based on the Kyoto Encyclopedia of Genes and Genomes (KEGG) database. All the statistical analyses were performed with SPSS Statistic 24.0 software (*p* < 0.05).

## 3. Results

### 3.1. Fruit Quality Analysis

We observed the effect of fruit quality on three late-maturation mandarin varieties grafted on four rootstocks for two consecutive years (Table 1). The results showed that fruit weight, fruit height, fruit diameter, fruit index, rind thickness, and titratable acid (TA) had no significant effects on the three varieties. Among the residual fruit quality indicators, the total soluble solid (TSS) content of ‘Gold Nugget’ and the TSS/TA and ascorbic acid contents of ‘Orah’ were different. The TSS content in the A1 treatment combination was the highest at 15.58%, and there was no significant difference in the TSS content among A2, A3, and A4. The TSS/TA of ‘Orah’ was the highest for ‘Carrizo citrange’, at 26.3%, and the lowest, at 18.5%, for ‘Red tangerine’. The TSS/TA ratio of ‘Gold Nugget’ on ‘Carrizo citrange’ reached 26.7%. The ascorbic acid content in the C3 treatment combination was the highest at 23.4 mg/100 mL, and the lowest level was observed in the C4 treatment combination. Figure 1 shows that rootstock had a relatively small impact on the basic quality indicators of Tango fruits, while multiple physiological indicators, including the TSS and TA contents, exhibited no significant differences among the different rootstocks. As a rootstock for the ‘Gold Nugget’, the trifoliate orange had a positive impact on the TSS content compared to that of the other three rootstocks. The rootstock variety significantly affected the TA content, and the sugar–acid ratio of ‘Orah’ and ‘Orah’ grafted on ‘Red Tangerine’ leaves had the highest TA content.

### 3.2. Metabolomic Profiling of Three Late-Maturation Mandarin Fruits with Different Rootstocks

**Figure 2:**
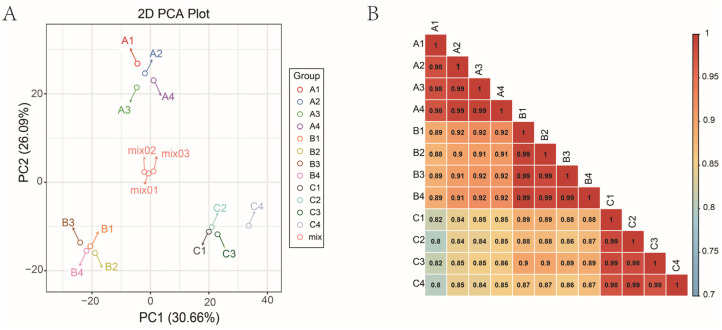
The principal component analysis (PCA) score (**A**) and correlation analysis (**B**).

A quality control (QC) sample was prepared by mixing sample extracts and used to analyze the repeatability of samples under the same treatment method. During instrumental analysis, one QC sample was inserted into every 10 test analysis samples to monitor the repeatability of the analysis process. The total ion current (TIC) plots and multipeak detection plots of one QC sample are shown in Figure S1. The overlap analysis of TIC plots of different QC samples showed that the curves of the total ion flow detected by metabolites had perfect overlap, and the retention time and peak intensity were consistent, which indicated that the signal stability was better when the same sample was detected by mass spectrometry at different times. The MRM metabolite detection multipeak plot shows the substances that can be detected in the samples, and each mass spectrum peak with a different color represents a metabolite detected (Figure S2). As shown in Figure 2A, ‘Gold Nugget’, ‘Tango’, and ‘Orah’ were clearly separated, and the same variety was compacted together and grafted on different rootstocks. These findings indicated that the test had good repeatability and reliability and that there were significant differences among the three varieties. The contribution rates of the two principal components reached 68.75%, with trends of 30.66% for PC1 and 28.09% for PC2. The R values in all the samples were greater than 0.8, suggesting that all the samples had a better correlation (Figure 2B).

**Figure 3:**
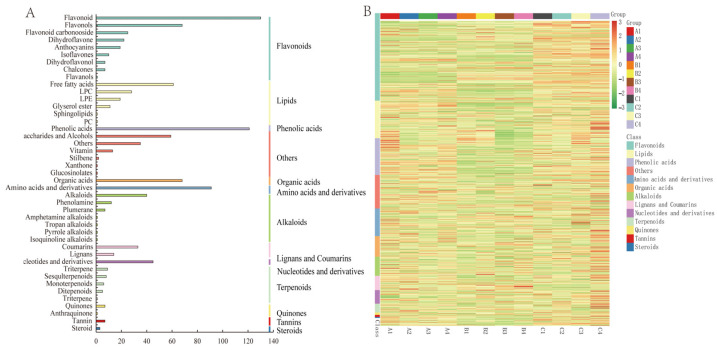
The number of different types of metabolites (**A**) and classification and clustering heatmaps of the metabolites (**B**) in all the samples.

Based on the local metabolic database, qualitative and quantitative mass spectrometry analyses of the metabolites were performed. A total of 1006 metabolites were identified in all rootstock–scion combinations (Table S1), including 13 major categories: flavonoids, lipids, phenolic acids, amino acids and derivatives, organic acids, alkaloids, lignans and coumarins, nucleotides and derivatives, terpenoids, quinones, tannins, steroids, and others. These metabolites were further subdivided into 13 subcategories in detail: 289 flavonoids (including 130 flavonoids, 68 flavonols, 7 halcones, 22 dihydroflavones, 25 flavonoid carbonosides, 7 dihydroflavones, 10 isoflavones, 1 flavanol, and 19 anthocyanins); 124 lipids (including 61 free fatty acids, 1 PC; 4 sphingolipids, 11 glycerol esters, 28 LPC, and 19 LPE); 121 phenolic acids; 91 amino acids and derivatives; 68 organic acids; 63 alkaloids (including 40 alkaloids, 12 phenolamines, 7 pluralones, 1 isoquinoline alkaloid, 1 pyrrole alkaloid, 1 tropan alkaloid, and 1 amphetamine alkaloid); 47 lignans and coumarins (including 14 lignans and 33 coumarins); 45 nucleotides and derivatives; 29 terpenoids (9 triterpenes, 1 terpene, 8 sesquiterpenoids, 6 monoterpenoids, and 5 diterpenoids; 8 quinones (including 7 quinones and 1 anthraquinone); 7 tannins; 3 steroids; and 111 others (59 saccharides and alcohols, 35 others, 13 vitamins, 2 stilbenes, 1 xanthone, and 1 glucosinolate) (Figure 3A). In addition, clustering heatmap analysis clearly revealed that these metabolites could be divided into 12 groups, suggesting that there were significant differences in metabolite content among the twelve scion–rootstock combinations. These results indicated that rootstock type affected the metabolic profiles of the different varieties (Figure 3B). As shown in the heatmap, the flavonoid content of ‘Orah’ was significantly greater than that of ‘Gold Nugget’ and ‘Tango’. Among them, when ‘Ziyang Xiangcheng’ was used as the rootstock, the contents of various metabolites were significantly greater than those of the other three types of rootstock. The content of phenolic acid in ‘Tango strawberry plants was significantly lower than that in ‘Gold Nugget’ and ‘Orah’, and it was greater than that in the other three types of rootstock when ‘Ziyang Xiangcheng’ was used as the rootstock. The lipid content of ‘Tango’ varied greatly among the different rootstocks. When ‘Tango’ was grafted on ‘Trifoliate orange’, the lipid content was the highest, followed by that of ‘Carrizo citrange’. Compared to that of ‘Carrizo citrange’, ‘Red tangerine’, and ‘Ziyang Xiangcheng’, when ‘Trifoliate orange’ was used as the rootstock, the lipid content of ‘Gold Nugget’ was greater, and ‘Carrizo citrange’ had the lowest lipid content.

### 3.3. Differential Metabolites Screening and Enrichment Analysis among Different Rootstock–Scion Combinations

**Figure 4:**
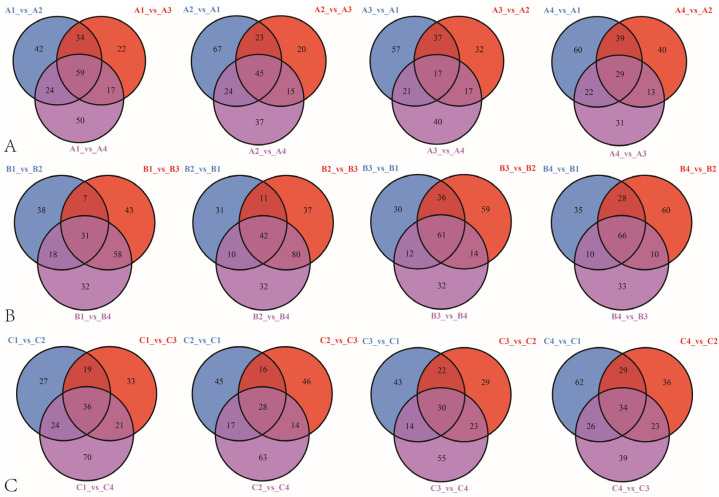
Venn diagram of the common differentially abundant metabolites in the comparison groups of the three mandarins grafted on the four rootstocks. (**A**), ‘Gold Nugget’ grafted on four rootstocks. (**B**), ‘Tango’ grafted on four rootstocks. (**C**), ‘Orah’ grafted on four rootstocks. A1: rootstock ‘Trifoliate orange’, A2: rootstock ‘Carrizo citrange’, A3: rootstock ‘Red tangerine’, and A4: rootstock ‘Ziyang Xiangcheng’.

To explore the metabolites of the different varieties grafted on the different rootstocks, the differentially abundant metabolites of all pairwise comparisons were screened using a fold change ≥2 or ≤0.5 and VIP ≥ 1 as the criteria (Table S2). According to Table S2, in the ‘Gold Nugget’, six sets of data (A1 vs. A2, A1 vs. A3, A1 vs. A4, A2 vs. A3, A2 vs. A4, and A3 vs. A4) were compared. Among them, 159 (93 downregulated and 66 upregulated) of the metabolites were significantly different between the A1 and A2 groups. According to Figure 4, 59 common differentially abundant metabolites were identified in the A1 vs. A2, A1 vs. A3, and A1 vs. A4 comparison groups, 22 of which were lipids. In the A2 vs. A1, A2 vs. A3, and A2 vs. A4 comparative groups; A3 vs. A1, A3 vs. A2, and A3 vs. A4 comparative groups; and A4 vs. A1, A4 vs. A2, and A4 vs. A3 comparative groups, 45, 17, and 29 differentially abundant metabolites were identified, respectively. Among them, lipids accounted for the largest proportion of lipids (20 out of 45) in the A2 vs. A1, A2 vs. A3, and A2 vs. A4 comparative groups. Flavonoids accounted for the largest proportion of the A3 vs. A1, A3 vs. A2, and A3 vs. A4 comparative groups, as well as in the A4 vs. A1, A4 vs. A2, and A4 vs. A3 control groups. In ‘Tango’, there were 170 significantly different metabolites (125 downregulated and 45 upregulated) in B2 vs. B3, and the proportion of lipids among these downregulated substances reached 48.80%. Flavonoids were the main differentially abundant metabolites in the B1 vs. B2, B1 vs. B3, and B1 vs. B4 groups and B2 vs. B1, B2 vs. B3, and B2 vs. B4 groups, accounting for 9 out of 31 and 18 out of 41, respectively. Lipids were the most diverse metabolites in the B3 vs. B1, B3 vs. B2, and B3 vs. B4 groups and B4 vs. B1, B4 vs. B2 and B4 vs. B3 groups, accounting for 40 out of 61 and 38 out of 66, respectively. In ‘Orah’, there were 151 significantly different metabolites between C1vs. C4 (48 downregulated and 103 upregulated). In the C4 vs. C1, C4 vs. C2, and C4 vs. C3 comparisons, lipids were the most common differentially abundant metabolites, occurring in 6 out of 34 samples. For the C1 vs. C2, C1 vs. C3, and C1 vs. C4 groups and C3 vs. C1, C3 vs. C2, and C3 vs. C4 groups, flavonoids were the group with the highest proportion of common differentially abundant metabolites, accounting for 8 out of 36 and 8 out of 30, respectively. Specifically, in the C2 vs. C1, C2 vs. C3, and C2 vs. C4 comparisons, the category with the highest proportion of common differentially abundant metabolites was phenolic acid, accounting for 6 out of 28. Moreover, in each control group, quinone compounds were present among the common differentially abundant metabolites only in the C1 vs. C2, C1 vs. C3, and C1 vs. C4 groups.

Overall, according to Table S2 and Figure 3, lipids and flavonoids were the main differentially abundant metabolites in our various comparison groups, whether for rootstocks or scions.

**Figure 5:**
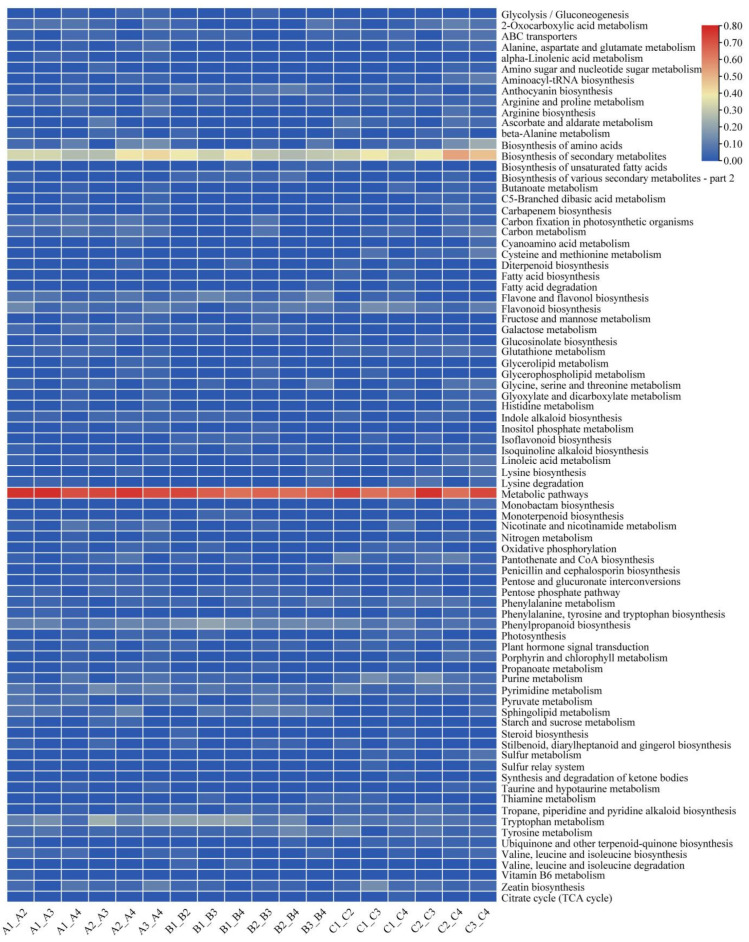
KEGG pathway analysis of differential metabolites in mandarin varieties.

The KEGG is a database resource for understanding high-level functions and utilities of biological systems, such as cells, organisms, and ecosystems, from genomic and molecular-level information. In this study, we enriched the differentially abundant metabolites of each comparison group (*p* value < 0.05). Among the ‘gold nugget’ A1 vs. A2, A1 vs. A3, A1 vs. A4, A2 vs. A3, A2 vs. A4, and A3 vs. A4 comparisons, the metabolites identified were involved mainly in 38, 24, 52, 31, 37, and 49 pathways, respectively, and the major pathways are presented in Table S3. According to Figure 5, the metabolic pathways related to ‘tryptophan metabolism’ were the most significantly enriched pathways in all the comparison groups except ‘A1 vs. A4’, followed by ‘sphingolipid metabolism’. In addition, the significantly enriched metabolic pathways identified in the comparisons of A1 vs. A2, A1 vs. A3, A1 vs. A4, A2 vs. A3, A2 vs. A4, and A3 vs. A4 were also related to ‘pyruvate metabolism’, ‘carbon fixation in photosynthetic organisms’, ‘phenylpropanoid biosynthesis’, and ‘zeatin biosynthesis’. In ‘Tango’, differentially abundant metabolites from all the comparison groups B1 vs. B2, B1 vs. B3, B1 vs. B4, B2 vs. B3, B2 vs. B4, and B3 vs. B4 were classified into 29, 23, 24, 35, 22, and 24 metabolic pathways, respectively (Figure 5, Table S3); ‘tryptophan metabolism’ and ‘sphingolipid metabolism’ were the major metabolic pathways among all the comparison groups and were similar to those of ‘Gold Nugget’. In ‘Orah’, differentially abundant metabolites from all the comparison groups C1 vs. C2, C1 vs. C3, C1 vs. C4, C2 vs. C3, C2 vs. C4, and C3 vs. C4 were classified into 29, 29, 51, 30, 47, and 51 metabolic pathways, respectively (Figure 5, Table S3). In contrast to those in the previous two varieties, the metabolic pathways in the present study were more diverse and related to ‘amino acid-related metabolism’, ‘pantothenate and CoA biosynthesis’, ‘zeatin biosynthesis’, ‘porphyrin and chlorophyll metabolism’, ‘sulfur metabolism’, etc.

### 3.4. Analysis of Metabolite Differences among the Three Late-Maturation Hybrid Mandarin Varieties

**Figure 6:**
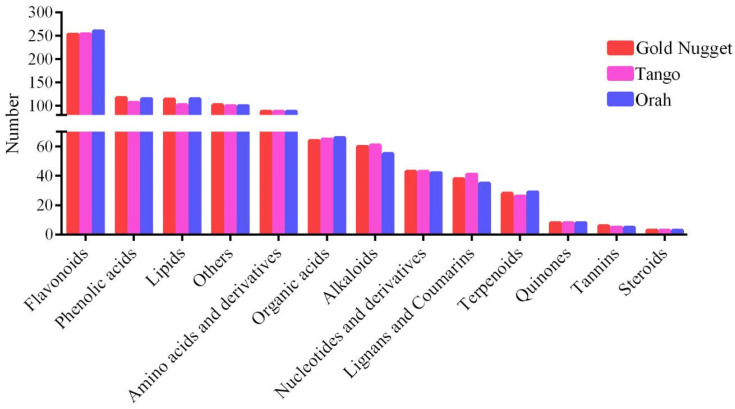
The number of metabolites identified in ‘Gold Nugget’, ‘Tango’ and ‘Orah’.

To understand the special metabolic characteristics of the three varieties, metabolites that were not detected simultaneously in each variety grafted on the four rootstocks were removed, and the numbers of metabolites identified as ‘Gold Nugget’, ‘Tango’, and ‘Orah’ were 924, 903, and 921, respectively (Table S4). These results indicated that the difference in metabolites detected may depend on the specific variety. Among the ‘Gold Nugget’, 960, 962, 958, and 965 metabolites were detected in ‘Trifoliate orange’, ‘Carrizo citrange’, ‘Red tangerine’, and ‘Ziyang Xiangcheng’, respectively. Among the metabolites detected in all the samples, 19 common metabolites were absent in the four rootstock treatment groups; these metabolites included 16 flavonoids, 1 terpenoid, 1 alkaloid and 1 other compound (Table S4). In ‘Tango’, 961, 968, 938, and 952 metabolites were identified from the four rootstocks, and 16 common metabolites were not found, including 7 flavonoids, 3 phenolic acids, 2 lignans and coumarins, 1 terpenoid, 1 tannin, and 2 others. Additionally, 958, 965, 949, and 961 metabolites were detected in ‘Orah’ grafted on the four rootstocks, and 17 common metabolites were absent, including 8 flavonoids, 1 phenolic acid, 3 lignans and coumarins, 3 alkaloids, 1 lipid, and 1 other metabolite. Detailed information is shown in Figure 6 and Table S5. Most importantly, four flavonoid metabolites, 5,7,4′-trihydroxy-8-methoxyflavone-6-C-[xylosyl-(1-2)]-glucoside), isosaponarin, luteolin-6-C-(5″-glucuronyl)xyloside, and apigenin-8-C-glucoside-7-O-sophoroside, were absent from both the ‘Gold Nugget’ and ‘Tango’. These findings suggest that the similar genetic backgrounds of ‘Gold Nugget’ and ‘Tango’ may lead to convergence of metabolic mechanisms.

### 3.5. Rootstocks Regulate Specific Metabolites in Fruits of Three Mandarin Varieties

**Figure 7:**
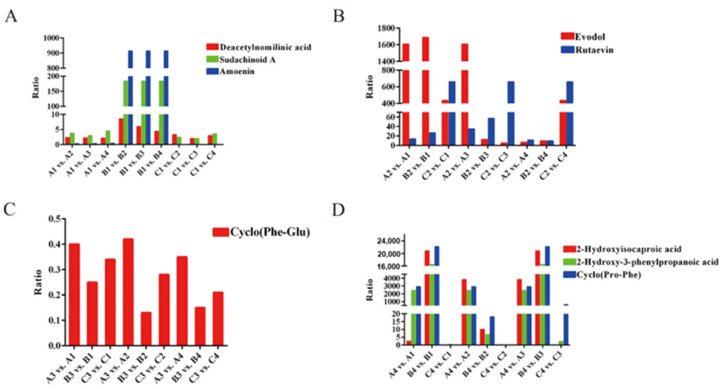
The metabolites were specifically regulated by the four different rootstocks. (**A**),‘Trifoliate orange’. (**B**), ‘Carrizo citrange’. (**C**), ‘Red tangerine’. (**D**), ‘Ziyang Xiangcheng’; Ratio: The relative ratio of metabolic data.

The rootstock affects the sugar, acid and flavor of scions of both fruits and vegetables. In this work, further analysis revealed that the levels of two metabolites, deacetylnomylinic acid and sudachinoid A, in the fruits of three varieties, obtained using ‘Trifoliate orange’ as rootstock were significantly greater than those obtained for the other three rootstocks, indicating that ‘Trifoliate orange’ may specifically promote the synthesis of deacetylnomylinic acid and sudachinoid A (Figure 7A, Table S6a). There was a difference in the flavonol metabolite amoAin. Compared with that in the other three rootstocks, the amoenin content in the fruits of ‘Gold Nugget’ and ‘Orah’ grafted on ‘Trifoliate orange’ was significantly lower, but the opposite was observed in ‘Tango’. As shown in Figure 7B and Table S6b, when Carrizo citrange was used as the rootstock, the contents of evodol and rutaevin were greater in the fruits of the three varieties than in those of the other rootstocks. Notably, ‘Red tangerine’ significantly inhibited cyclo(phenylalanine-glutamic acid) accumulation because it had the lowest content, approximately 0.13~0.42 of that in the three varieties grafted on the other rootstocks (Figure 7C, Table S6c). In addition, the cyclo(proline-phenylalanine) 2-hydroxyisocaproic acid and 2-hydroxy-3-phenylpropanoic acid contents in the fruits of ‘Gold Nugget’ and ‘Tango’ fruits generated using ‘Ziyang Xiangcheng’ as rootstock were significantly greater than those in the fruits of the other rootstocks. However, the 2-hydroxyisocaproic acid content of ‘Orah’ fruit grafted on ‘Ziyang Xiangcheng’ was markedly lower than that of the other plants, while the 2-hydroxy-3-phenylpropanoic acid and cyclo (proline-phenylalanine) levels were significantly greater than those of ‘Trifoliate orange’ and ‘Carrizo citrange’, except for ‘Red tangerine’ (Figure 7D, Table S6d). These findings suggest that rootstocks may have a bias in regulating fruit metabolites among the special metabolic pathways of the three varieties.

## 4. Discussion

Rootstock selection has a direct impact on fruit quality, yield, and tree growth, and ultimately leads to differences in fruit appearance quality and internal quality [28,29,30]. These differences are often determined by metabolic products. Many studies have shown that rootstock treatment directly affects the metabolic profile of fruits and even leaves [31]. Studies of fruits via metabolomics can reveal changes in metabolites and clarify the relationship between metabolites and phenotypes, including important metabolites related to sensory quality and nutrition, to further guide quality improvement breeding. Therefore, exploring the influence of rootstock on the metabolites produced by different citrus fruits is highly important for improving citrus quality and yield. Studying the effect of rootstock on scion growth parameters is highly important for improving fruit cultivation [32]. In the present study, fruit quality analysis of three mandarin varieties grafted onto four rootstocks was carried out over two successive years, and the results revealed that rootstock had an obvious influence on the fruit quality indices of the three varieties. Among them, the TSS content of the fruits of ‘Gold Nugget’ on ‘Trifoliate orange’ was significantly greater than that of the fruits of the other three rootstocks, indicating better quality. This result is similar to previous research results [12,13].

In this work, a total of 1006 metabolites were identified, comprising 13 categories. The greatest number of metabolites was flavonoids (289), which included flavonoids, flavonols, flavonoid carbonosides, dihydroflavones, and anthocyanins, followed by 124 lipids, 121 phenolic acids, and 111 others. This result is similar to that of previous research, in which flavonoids were the main metabolites found in citrus [33], while hesperidin was the main flavonoid compound [34]. Flavonoids, important secondary compounds in plants, participate in physiological processes, such as the attraction of pollinators to petals, interactions with microorganisms, pollen fertility and germination [35]. In addition, flavonoid metabolites have shown important protective biological activities, including reducing the risk of cardiovascular disease, cancer, and chronic diseases [36]. Citrus flavanone metabolites are the main source of total flavonoids consumed in the U.S. and Brazilian diets, and can protect pancreatic β cells [37]. Therefore, flavonoids play an important role in both the plants themselves and the humans who consume their fruits.

Through PCA and heatmap analysis, it was clear that there were significant differences in the metabolic products produced by the different combinations of rootstocks and scions, and the products were specific to some extent because of the metabolic accumulation characteristics of the rootstocks and scions. These characteristics could be used as powerful indicators of family evolution [38]. Vaclavik et al. successfully identified orange juice mixed with 15% apple juice and grapefruit juice through metabolomic fingerprint analysis [39]. In this study, we found that rootstock mainly affected the metabolism of flavonoids, phenolic acids, and lipids in the three varieties. The differentially abundant metabolites of fruits, lipids and flavonoids in ‘Gold Nugget’; flavonoids, alkaloids, lignans, and coumarins in ‘Tango’; and flavonoids and phenolic acids in ‘Orah’ might explain the differences in taste and quality among the different rootstocks. In addition to affecting the taste of fruits, rootstocks can also regulate the biosynthesis of various compounds, such as amino acids [40], phenolic acids [41], alkaloids [42], and flavonoids [43], and improve plant tolerance to various stresses by regulating the biosynthesis of metabolic products. Rootstocks can affect the tolerance of citrus scions to pathogens by affecting primary and secondary metabolites [44], and can also improve the low-temperature resistance of bitter gourd by regulating sucrose and nitrogen metabolism [45]. These results all indicate that different rootstock–scion combinations have different metabolic profiles, which in turn result in phenotypic differences.

KEGG pathway enrichment analysis helps to elucidate the mechanism underlying the changes in metabolic pathways. In ‘Gold Nugget’, KEGG pathway analysis revealed that differentially abundant metabolites were significantly enriched in sphingolipid metabolism, pyruvate metabolism, tryptophan metabolism, and carbon fixation in photosynthetic organisms. Sphingolipids, major lipids, are essential metabolites in all plant species and are bioactive metabolites that regulate cell function [46]. Pyruvic acid is a key intermediate in glucose metabolism and is involved in the mutual transformation of sugars, lipids and amino acids through acetyl-CoA and the tricarboxylic acid cycle. Tryptophan is an important nutrient that is also a precursor of many growth regulators and some secondary metabolites in plants, such as IAA, glucosinolates, and camalexin. It was found that spraying tryptophan could improve the growth and yield of plants [47]. Carbon fixation in photosynthetic organisms could provide energy substances for life. In ‘Tango’, differentially abundant metabolites were significantly enriched in the pathways of tryptophan metabolism, anthocyanin biosynthesis, and phenylpropanoid biosynthesis. Anthocyanins are involved in many aspects of plant development and defense, and they are synthesized by the flavonoid biosynthetic pathway through the phenylalanine pathway [48]. Phenylpropanoids are precursors of lignins and have structural functions, antioxidant activities, and drought tolerance [49]. Phenylpropanoid biosynthesis is the most critical pathway for the synthesis of characteristic phenolic metabolites [50]. In ‘Orah’, the differentially abundant metabolites were significantly involved in pantothenate and CoA biosynthesis, zeatin biosynthesis, purine metabolism, photosynthesis, and oxidative phosphorylation. Pantothenate acids, also known as vitamin B5, are precursors of CoA and ACP, and mainly affect fatty acid metabolism. Purine metabolism, photosynthesis, and oxidative phosphorylation could provide energy for plant growth and development. In brief, the above pathways might be related to the differences in taste and quality among the three varieties on the different rootstocks. Furthermore, the mechanisms of the enrichment of differentially abundant metabolites in the KEGG pathway need to be further explored.

Previous studies have shown that hesperetin and its derivatives are characteristic flavanones of sweet orange, tangelo, lemon, and lime, while naringenin and its derivatives are characteristic of grapefruit and sour orange [51]. An analysis of flavonoid metabolites of citrus peels revealed that the tricin 40-O-syringyl alcohol of Dahongpao could be used as a marker to distinguish other varieties [33]. Nine metabolites, including didymin, rhoifolin, isorhoifolin, neohesperidin, hesperidin, naringin, narirutin, limonin glucoside, and vicenin-2, were identified as the main markers and could be used for identification of adulteration in Indian citrus fruits/fruit juices [52]. In this work, we found that nine differentially abundant metabolites (deacetylnomilinic acid, sudachinoid A, amoenin, evodol, rutaevin, cyclo (phenylalanine-glutamic acid), 2-hydroxyisocaproic acid, 2-hydroxy-3-phenylpropanoic acid, and cyclo (proline-phenylalanine)) of three varieties were specifically regulated by different rootstocks. In conclusion, the results obtained in the present study highlight the special influence of rootstock on fruit metabolites while also providing a theoretical basis for the optimization of rootstockck–scion combinations in citrus production. This study selected only three varieties for analysis. Although these nine differential metabolic components can be regulated to some extent by the four rootstocks, further verification through the analysis of additional varieties is needed to determine whether these substances are marker metabolites of the rootstock. 

The specific metabolites found in this study are relatively novel substances, and there is no standard substance for accurate quantitative determination of these substances. Therefore, in the follow-up study, we will use new chemical analysis technology to separate and purify these specific substances, identify their structural characteristics, and synthesize and prepare their standard products, so as to facilitate the quantitative verification analysis of these substances in more varieties. In recent years, a number of multi-omics analyses such as transcriptomics, proteomics, and metabolomics have been used to study rootstock-mediated effects involved in the modification of gene expression and secondary metabolites in plants, including citrus, grapevine, and apple [7,53,54,55,56]. Hence, in future research endeavors, transcriptomics and proteomics analyses will be utilized to explore the key genes regulating the synthesis of these substances, and to analyze the molecular mechanisms that contribute to variations in metabolite content among rootstocks.

## 5. Conclusions

In summary, the three mandarin varieties had similar metabolite compositions but with different metabolite numbers. Flavonoids and phenolic acids are the most important compounds in citrus fruits. Considerable differences among the different rootstocks were also observed in the accumulation of phenolic acids, flavonoids, and lipids. KEGG analysis indicated that rootstock significantly affects metabolic pathways related to tryptophan metabolism, phenylpropanoid biosynthesis, sphingolipid metabolism, and pantothenate and CoA biosynthesis. Among the tested rootstocks, ‘Trifoliate orange’ significantly enhanced the deacetylnomylinic acid and sudachinoid A contents of citrus fruits, while the evodol and rutaevin contents of three varieties of fruit grafted onto ‘Carrizo citrange’ dramatically increased, and ‘Ziyang Xiangcheng’ had great potential to significantly increase the organic acid content (2-hydroxyisocaproic acid, 2-hydroxy-3-phenylpropanoic acid). However, the level of cyclo (phenylalanine-glutamic acid) significantly decreased when ‘Red tangerine’ was used as the rootstock. The results of this study provide new insight into rootstock selection for citrus cultivars.

## Figures and Tables

**Table 1:** Effects of four rootstocks on the fruit quality of three late-maturation mandarins.

Variety	Rootstock	Fruit Weight (g)	Fruit Height (cm)	Fruit Diameter (cm)	Fruit Index	Rind Thickness (mm)	TSS (%)	TA (‰)	TSS/TA	Ascorbic Acid (mg/100 mL)
**Gold Nugget A**	1	97.2 ± 10.1 a	4.8 ± 0.2 a	6.1 ± 0.3 a	0.79 a	3.5 ± 0.3 a	15.6 ± 0.2 a	7.2 ± 0.2 a	21.9 ± 0.5 a	14.9 ± 0.3 a
	2	116.9 ± 8.3 a	5.2 ± 0.2 a	6.5 ± 0.1 a	0.81 a	3.8 ± 0.2 a	13.7 ± 0.1 b	5.2 ± 0.2 a	26.7 ± 0.3 a	14.8 ± 0.2 a
	3	118.5 ± 5.3 a	5.2 ± 0.1 a	6.6 ± 0.4 a	0.80 a	3.8 ± 0.2 a	13.5 ± 0.2 b	5.8 ± 0.1 a	23.5 ± 0.4 a	13.2 ± 0.3 a
	4	105.9 ± 7.2 a	4.9 ± 0.3 a	6.4 ± 0.2 a	0.78 a	4.2 ± 0.3 a	13.5 ± 0.1 b	5.8 ± 0.1 a	23.5 ± 0.2 a	14.4 ± 0.6 a
**Tango** **B**	1	87.0 ± 5.7 a	4.7 ± 0.2 a	6.0 ± 0.2 a	0.78 a	3.1 ± 0.1 a	12.3 ± 0.2 a	7.9 ± 0.3 a	15.8 ± 0.2 a	22.3 ± 0.7 a
	2	98.3 ± 6.9 a	4.8 ± 0.3 a	6.2 ± 0.3 a	0.78 a	2.9 ± 0.1 a	11.8 ± 0.1 a	7.4 ± 0.2 a	16.1 ± 0.3 a	20.7 ± 0.5 a
	3	90.9 ± 8.1 a	4.7 ± 0.3 a	6.0 ± 0.3 a	0.78 a	2.8 ± 0.2 a	12.1 ± 0.0 a	7.6 ± 0.1 a	15.9 ± 0.5 a	22.5 ± 0.1 a
	4	88.17 ± 4.8 a	4.7 ± 0.1 a	5.9 ± 0.4 a	0.80 a	3.1 ± 0.3 a	12.3 ± 0.2 a	7.1 ± 0.2 a	17.4 ± 0.4 a	22.5 ± 0.3 a
**Orah** **C**	1	116.3 ± 8.5 a	5.2 ± 0.2 a	6.4 ± 0.3 a	0.81 a	3.8 ± 0.1 a	14.2 ± 0.3 a	6.1 ± 0.3 a	23.6 ± 0.3 ab	19.6 ± 0.2 ab
	2	127.5 ± 7.9 a	5.4 ± 0.3 a	6.6 ± 0.4 a	0.82 a	4.0 ± 0.1 a	13.5 ± 0.3 a	5.1 ± 0.1 a	26.3 ± 0.5 a	21.0 ± 0.4 ab
	3	89.4 ± 4.0 a	4.9 ± 0.2 a	5.8 ± 0.1 a	0.83 a	3.7 ± 0.2 a	14.0 ± 0.1 a	7.9 ± 0.2 a	18.5 ± 0.2 b	23.5 ± 0.3 a
	4	119.5 ± 6.5 a	5.3 ± 0.5 a	6.4 ± 0.3 a	0.82 a	4.3 ± 0.1 a	13.5 ± 0.2 a	5.4 ± 0.3 a	25.4 ± 0.6 ab	17.7 ± 0.3 b

Note: The data in the table are two-year average values, different letters above the bars on columns show a significant difference at *p* < 0.05.

## Data Availability

The original contributions presented in the study are included in the article, further inquiries can be directed to the corresponding author/s.

## Supplementary Materials

The following supporting information can be downloaded at: https://www.mdpi.com/article/10.3390/metabo14040242/s1, Figure S1: QC MS tic overlap; Figure S2: MRM detection of multimodal maps; Table S1: Metabolic data of all samples; Table S2: Statistical table of differential metabolites for pairwise comparison of all combinations; Table S3: The cluster frequency analyses of KEGG pathway in mandarin varieties; Table S4: Identified metabolites actually belong to ‘Gold Nugget’, ‘Tango’, ‘Orah’; Table S5: Common metabolites absent in three varieties grafted on different rootstocks; Table S6: Specific regulated metabolites based on different rootstocks.

## Appendix A

**Table A1:** Abbreviation correspondence table.

No.	Full Meaning	Abbreviation
1	Ultra-performance liquid chromatography–tandem mass spectrometry	UPLC-MS/MS
2	Huanglongbing	HLB
3	Total soluble solids content	TSS
4	Titratable acidity	TA
5	Ascorbic acid	Vc
6	Curtain gas	CUR
7	Hierarchical cluster analysis	HCA
8	Unit variance scaling	UV
9	Principal component analysis	PCA
10	Kyoto Encyclopedia of Genes and Genomes	KEGG
11	Quality control sample	QC
12	Total ion current	TIC
