# Supplementary figures and images for: Widely Targeted Metabolomic Analysis Provides New Insights into the Effect of Rootstocks on Citrus Fruit Quality
Source: Metabolites. 2024 Apr 21;14(4):242. doi: 10.3390/metabo14040242 (PMC11052146; doi:10.3390/metabo14040242)

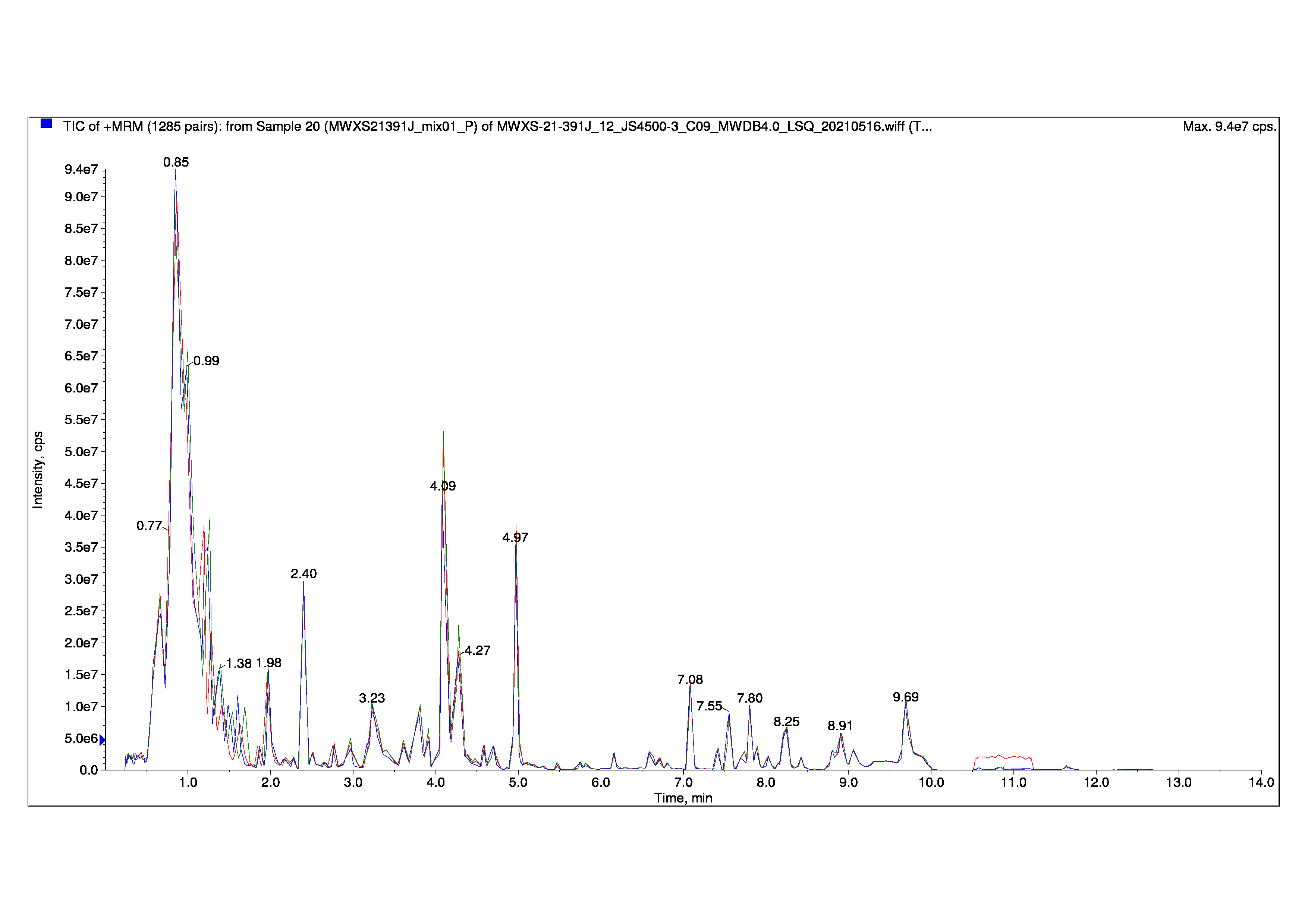

Supplement: Supplementary file 1 [file metabolites-14-00242-s001.zip › Supplementary Materials/Fig. S1ú║QC MS tic overlap-P.png]

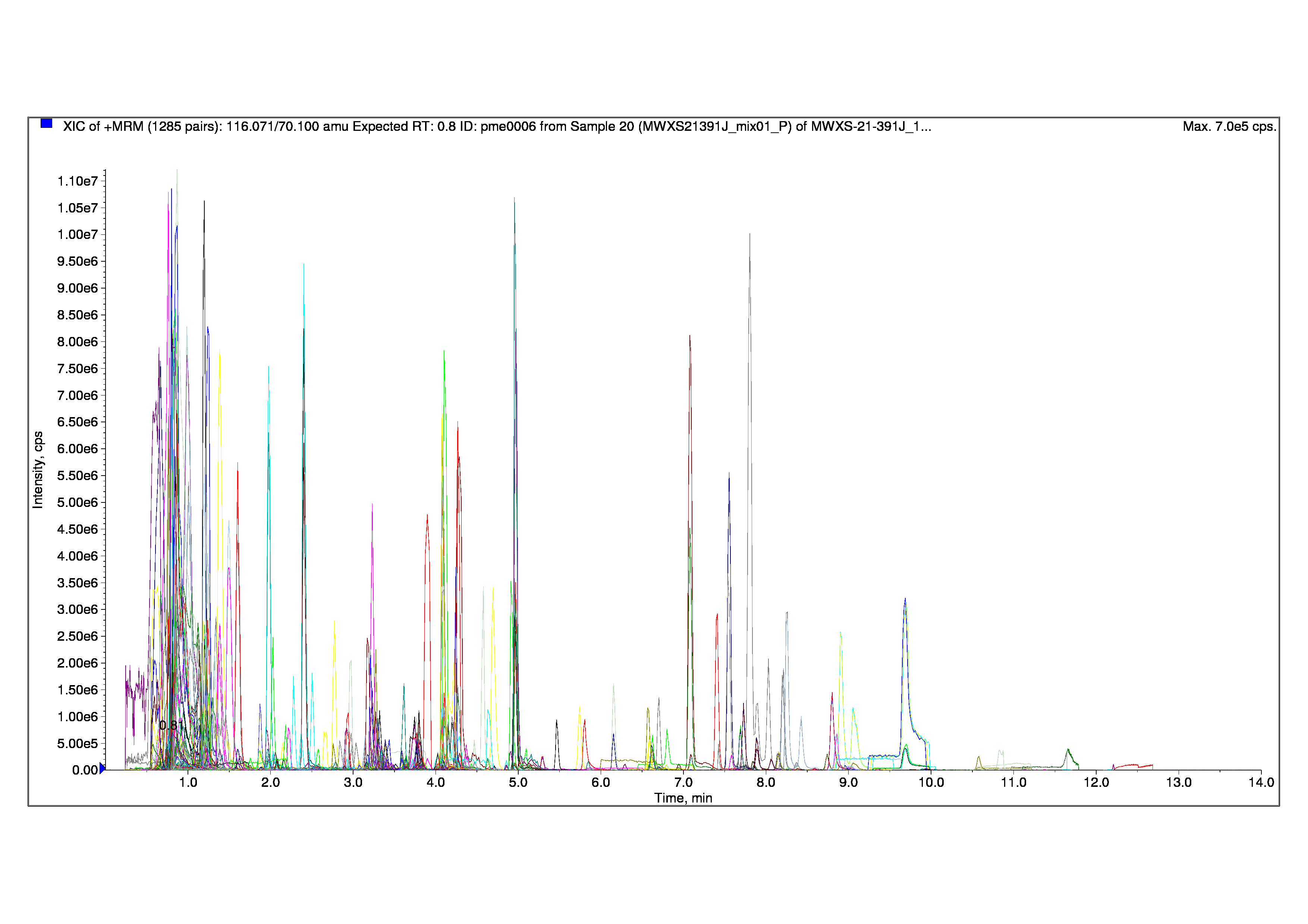

Supplement: Supplementary file 1 [file metabolites-14-00242-s001.zip › Supplementary Materials/Fig. S2ú║MRM detection of multimodal maps-P.png]
